# Study protocol for “Study of Practices Enabling Implementation and Adaptation in the Safety Net (SPREAD-NET)”: a pragmatic trial comparing implementation strategies

**DOI:** 10.1186/s13012-015-0333-y

**Published:** 2015-10-16

**Authors:** Rachel Gold, Celine Hollombe, Arwen Bunce, Christine Nelson, James V. Davis, Stuart Cowburn, Nancy Perrin, Jennifer DeVoe, Ned Mossman, Bruce Boles, Michael Horberg, James W. Dearing, Victoria Jaworski, Deborah Cohen, David Smith

**Affiliations:** Kaiser Permanente Northwest Center for Health Research, 3800 N. Interstate Avenue, Portland, OR 97227 USA; OCHIN, Inc., 1881 SW Naito Parkway, Portland, OR 97201 USA; Oregon Health Science University, 3181 S.W. Sam Jackson Park Rd., Portland, OR 97239 USA; Kaiser Permanente Care Management Institute, 1 Kaiser Plaza, 16 L, Oakland, CA 94612 USA; Mid-Atlantic Permanente Research Institute, 2101 East Jefferson Street 3 West, Rockville, MD 20852 USA; College of Communication Arts and Sciences, Michigan State University, 404 Wilson Road, 473, East Lansing, MI 48824 USA; Multnomah County Public Health Department, 426 SW Stark St, 8th Floor, Portland, OR 97204 USA

**Keywords:** Diabetes mellitus, Cardiovascular disease, Quality improvement, Community health centers, Evidence-based, Translational medical research

## Abstract

**Background:**

Little research has directly compared the effectiveness of implementation strategies in any setting, and we know of no prior trials directly comparing how effectively different combinations of strategies support implementation in community health centers. This paper outlines the protocol of the Study of Practices Enabling Implementation and Adaptation in the Safety Net (SPREAD-NET), a trial designed to compare the effectiveness of several common strategies for supporting implementation of an intervention and explore contextual factors that impact the strategies’ effectiveness in the community health center setting.

**Methods/design:**

This cluster-randomized trial compares how three increasingly hands-on implementation strategies support adoption of an evidence-based diabetes quality improvement intervention in 29 community health centers, managed by 12 healthcare organizations. The strategies are as follows: (arm 1) a toolkit, presented in paper and electronic form, which includes a training webinar; (arm 2) toolkit plus in-person training with a focus on practice change and change management strategies; and (arm 3) toolkit, in-person training, plus practice facilitation with on-site visits. We use a mixed methods approach to data collection and analysis: (i) baseline surveys on study clinic characteristics, to explore how these characteristics impact the clinics’ ability to implement the tools and the effectiveness of each implementation strategy; (ii) quantitative data on change in rates of guideline-concordant prescribing; and (iii) qualitative data on the “how” and “why” underlying the quantitative results. The outcomes of interest are clinic-level results, categorized using the Reach, Effectiveness, Adoption, Implementation, Maintenance (RE-AIM) framework, within an interrupted time-series design with segmented regression models. This pragmatic trial will compare how well each implementation strategy works in “real-world” practices.

**Discussion:**

Having a better understanding of how different strategies support implementation efforts could positively impact the field of implementation science, by comparing practical, generalizable methods for implementing clinical innovations in community health centers. Bridging this gap in the literature is a critical step towards the national long-term goal of effectively disseminating and implementing effective interventions into community health centers.

**Trial registration:**

ClinicalTrials.gov, NCT02325531

## Background

The adoption and implementation of evidence-based care guidelines and quality improvement (QI) practices into everyday practice is limited; hence, scientific advances rarely reach their population potential in a timely manner [1–12]. One underlying reason is that most prior research focused on developing and evaluating interventions but not on the distinct methods used to support such interventions’ uptake into practice [13–15]. As a result, there is incomplete knowledge about what strategies best support implementing proven innovations [2–9]. Broadly defined, “implementation strategies” may include the following: policies to incentivize intended users to adopt targeted innovations, providing treatment guidelines, decision support, training, consultation, facilitation, and/or feedback data; and using QI approaches such as workflow redesign, Plan-Do-Study-Act cycles, and other change management practices [16–18].

The Study of Practices Enabling Implementation and Adaptation in the Safety Net (SPREAD-NET) builds on existing knowledge on the efficacy of different strategies for implementing interventions that seek to improve clinical outcomes by changing clinician behaviors. Academic detailing (expert consultation)/educational outreach are generally effective at changing provider behaviors, other process measures, and clinical outcomes [19–23]. Interactive small group trainings have a modest positive effect on provider performance [24–26]. In-person training and “train-the-trainer” approaches are supported by substantial evidence [8]. Toolkits can support implementation of guideline-based care [27], but “passive” implementation—i.e., simply providing a toolkit—shows mixed effectiveness [19]. “Auditing” and giving feedback data can change provider behaviors and other process measures but have mixed effects on clinical outcomes [28–38]. Practice facilitation (when skilled individuals help clinic staff implement interventions) [39, 40] can support enhanced care quality—for example, primary care practices are 2.76 (95 % CI, 2.18–3.43) times more likely to adopt guidelines if practice facilitation is used [39] but has mixed effects on clinical outcomes [39–41].

**Fig. 1:**
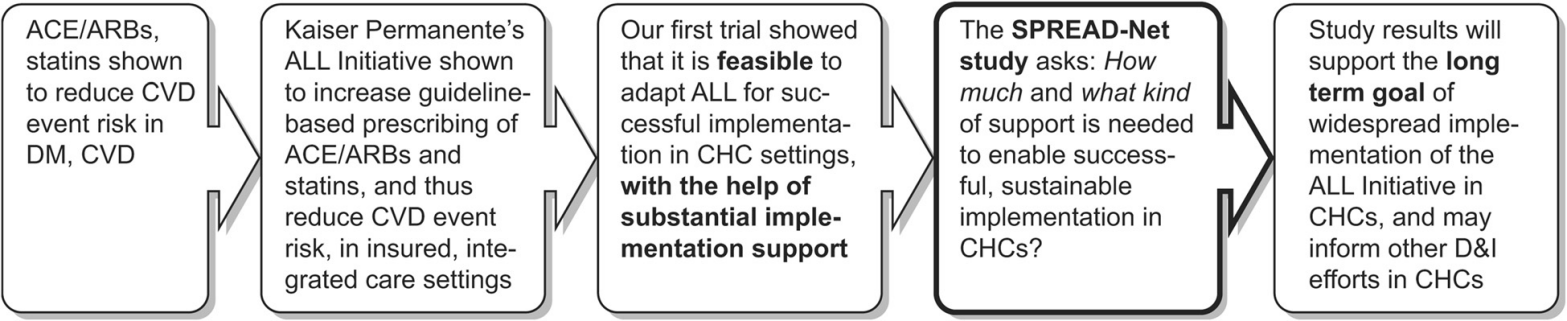
The SPREAD-NET study in the context of a larger line of research on improving cardiovascular outcomes in socioeconomically vulnerable patients with diabetes

As the most prior research studied one implementation strategy at a time, little is known about the benefits of multiple, concurrent implementation strategies (e.g., training plus technical assistance). Further, little research has directly compared the effectiveness of different implementation strategies in any setting [16, 17, 42, 43], much less in Community Health Centers (CHCs) [2], so it is unknown which individual or combined strategies—even those that work in more controlled research settings or private/academic settings—will work in CHCs. This is problematic, because CHCs serve millions of socioeconomically vulnerable patients in the USA [43–47]. In addition, little research has been conducted on the influence of context on the effectiveness of various implementation strategies [31]—i.e., which strategies are optimal in which settings and why [48, 49]. We believe that the “SPREAD-NET” trial is the first to compare the effectiveness of common strategies for supporting implementation of a proven QI intervention in CHCs. Figure 1 illustrates how this study fits within a line of inquiry on improving care quality in CHCs.

### The original intervention and implementation strategies

Kaiser Permanente (KP), a large integrated health care delivery system, developed the “A.L.L. Initiative” (aspirin, lovastatin (any statin), lisinopril (any angiotensin-converting-enzyme (ACE) inhibitor or angiotensin receptor blocker (ARB)), hereafter called “ALL”). ALL is a system-level QI intervention designed to increase the percentage of patients with cardiovascular disease (CVD)/diabetes mellitus (DM) taking cardioprotective medications according to national treatment guidelines [50]. At KP, the ALL intervention uses electronic health record (EHR) reminders and panel management tools to help providers identify patients indicated for but not taking an ALL medication. The ALL implementation strategies used at KP involve incentivizing providers to appropriately prescribe the ALL medications (via pay bonuses related to overall care quality) and directives identifying it as KP’s standard of care. At KP, ALL led to an estimated > 60 % reduction in CVD events among targeted adults [50].

We demonstrated the feasibility of implementing ALL in CHCs and its effectiveness in this population in our prior trial [NCT02299791], using ALL to test cross-setting translational implementation [42, 51]. As implemented, the intervention improved guideline-concordant prescribing with relative increases of almost 40 % (previously reported) [42]. We used multiple, concurrent strategies (including staff training, on-site facilitation, and performance reports) to support implementing ALL in 11 CHCs. Some CHCs used more intensive support (e.g., multiple staff trainings, repeated facilitation visits), others less ("just give us the EHR tools"). Though we showed such implementation to be feasible in CHCs, we wanted to understand how much and what type of implementation support would be needed to efficiently obtain similar success in other CHCs. Understanding this is relevant to future dissemination of ALL specifically and also provides general and generalizable knowledge to inform dissemination of other evidence-based interventions in CHCs. This purpose, and the fact that implementation strategies are largely understudied in CHCs, [8, 52–55] underlie the SPREAD-NET trial.

SPREAD-NET seeks to identify the specific strategies associated with successfully implementing and sustaining ALL in CHCs and the clinic characteristics associated with implementation success with different levels/types of support. To that end, its aims are as follows:

- Aim 1: Compare the effectiveness of three implementation strategies at supporting CHCs’ implementation of the ALL intervention, through a cluster-randomized trial.
- H1: Clinics randomized to receive more implementation support will be more likely than those randomized to receive less support (high>medium>low) to significantly improve the percent of their patients with (i) guideline-appropriate prescriptions for ACE/ARBs and statins and (ii) last blood pressure (BP) and low-density lipoprotein (LDL) under control. H1a. A minimum amount of support can be identified for effective, sustainable implementation of the intervention. H1b. The minimum support needed will differ between clinics with different characteristics.
- Aim 2: Assess intervention sustainability at 12, 24, and 36 months post-implementation.
- H2: Clinics receiving more implementation support will be more likely to maintain changes.
- Aim 3: Identify clinic characteristics associated with the strategies’ effectiveness (e.g., decision-making structures, leadership support, team processes/characteristics, readiness/capacity for change).

## Methods

### Study design

This cluster-randomized trial will compare how three increasingly hands-on strategies support implementation of ALL in “real world” CHCs. We use a concurrent mixed methods approach [56] to identify how and why outcomes are achieved across CHCs, *i.e.*, identifying successful support strategies, users’ perceptions of the strategies, and clinic factors that affect success. This work includes collaborators from the Kaiser Permanente NW Center for Health Research, OCHIN Inc., Oregon Health & Science University, Kaiser Permanente Mid-Atlantic States, Michigan State University, and the participating CHCs. Approval was obtained from the Kaiser Permanente NW Institutional Review Board.

### Study setting

Twenty-nine CHCs, managed by 12 healthcare organizations, are participating. These CHCs share a single instance of the EpicCare© EHR through membership in OCHIN, Inc., a non-profit health information technology organization [57–59]. OCHIN is a national collaborative providing health information technology to CHCs, with >400 member primary care clinics in 19 states. OCHIN members share a single, fully integrated, centrally hosted Epic© EHR wherein patients have an unduplicated, network-wide medical record [60, 61]. OCHIN also develops EHR-based decision support and panel management tools, directed by a member-led advisory group, the Clinical Operations Review Committee (CORC).

### Updating the ALL intervention

**Fig. 2:**
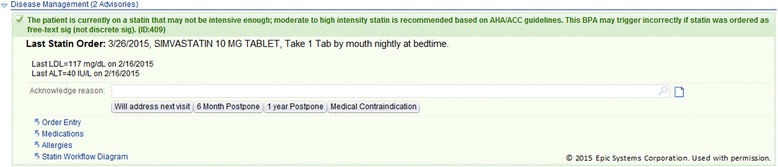
Alert noting that the patient’s statin dose is too low
- BPAs that promote accurate charting. For example, if a patient has high blood pressure but hypertension is not on their EHR problem list, an alert suggests adding it. If the patient had a relevant prescription that had not been renewed in over a year, an alert recommends updating the prescription or the chart. These “charting alerts” serve a dual purpose because the algorithms underlying the bundle are premised on charting accuracy. Figure 3 shows a BPA which notes that the patient’s statin prescription may be out of date.

We adapted the ALL intervention from how it was presented in our earlier translational study, to align it with updated national guidelines [62–68] and with concurrent QI activities at OCHIN. To that end, ALL-related components were added to a QI “bundle” that OCHIN’s CORC built for their shared EHR. Designed to address multiple aspects of DM/CVD care, this set of clinical decision support (CDS) tools is called the CVD risk management bundle (hereafter, the CVD bundle) and includes the following:

- “Best practice alerts” (BPAs) that activate in the EHR when a patient is indicated for an ACE/ARB and/or a statin, per current guidelines, but has no prescription for the medication(s). Per these guidelines, the BPAs also note if patients are prescribed the recommended statin dosage. Figure 2 shows a BPA saying the patient is on statin but is clinically indicated for a higher intensity dose.

**Fig. 3:**
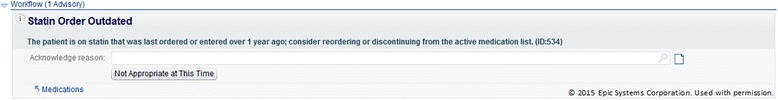
Alert noting that the patient’s statin order is outdated
- Roster/panel management tools that enable sorting complex patient population data. For example, they can be used to create lists for “scrubbing” incoming patients (reviewing the chart before patient visits to alert the team to needed care) [69] or for targeted outreach or to create performance feedback reports, as well as longitudinal data useful for QI reporting. Figure 4 shows the roster/panel management tool; Fig. 5 shows what the roster tool’s list for “scrubbing” incoming patients.

**Fig. 4:**
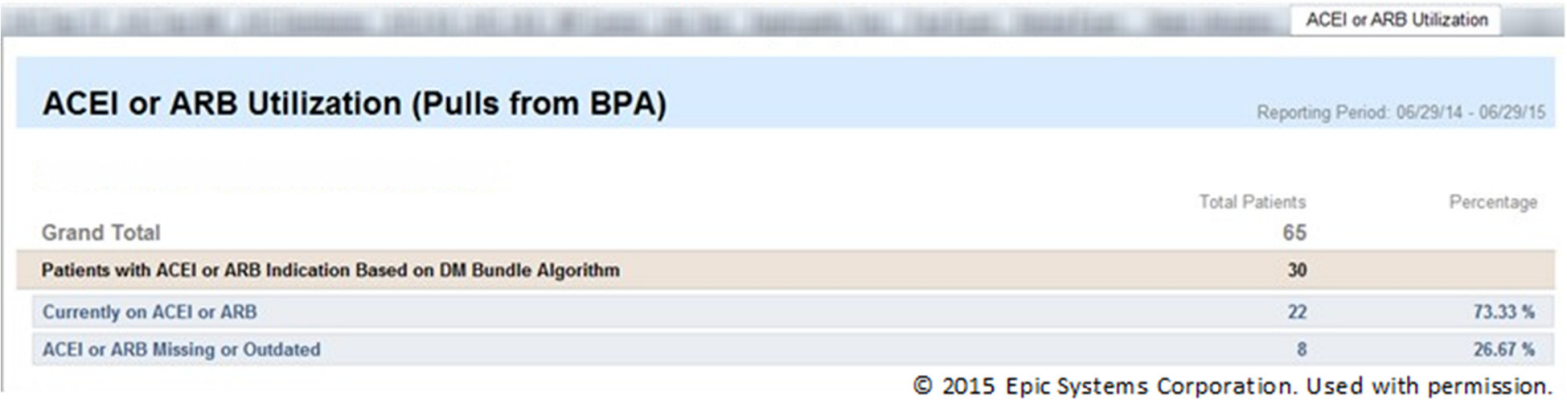
The roster/panel management tool

**Fig. 5:**
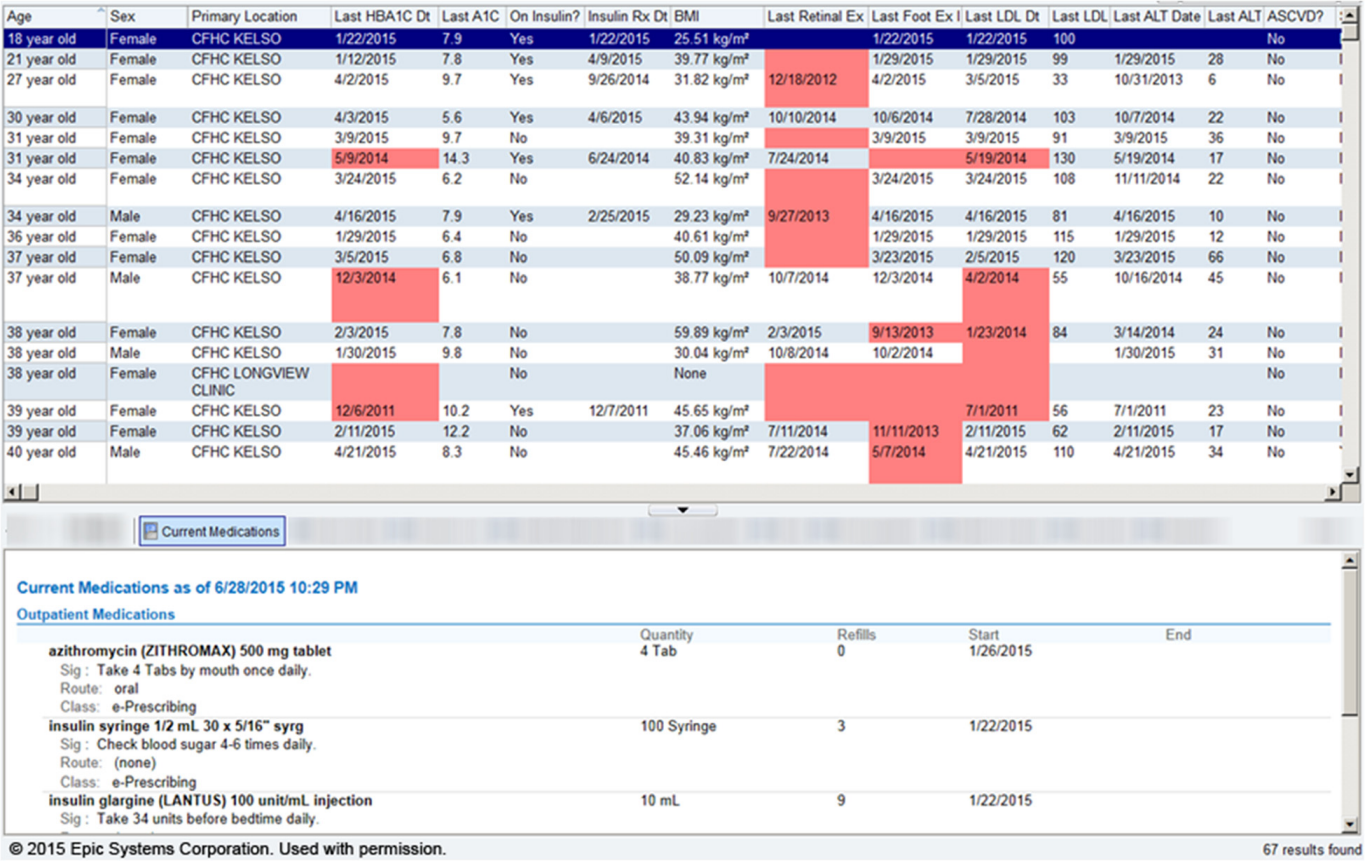
The roster tool’s list for “scrubbing” incoming patients
- “Health maintenance alerts” that activate when the chart is opened for a patient who is overdue for DM-related procedures (e.g., foot exams, HgbA1c screening).

### Timeline overview

In May 2014–July 2015, we updated the ALL intervention to represent current guidelines, helped OCHIN’s CORC to embed the ALL components in the CVD Bundle, developed and fine-tuned the implementation support strategies, randomized the participating CHCs to different study arms (each arm receiving a different level of implementation support), and conducted baseline surveys. In May 2015, the CVD Bundle went live at all OCHIN clinics. In July 2015, the study CHCs began receiving the implementation support to which they were randomized. Follow-up will last 3 years.

### Implementation support strategies

The standard practice when OCHIN implements QI bundles is to activate the associated EHR tools, make brief instructions for their use and explanations of their logic available on the OCHIN member website, and e-mail members to alert them that new tools are in place. In this case, two 90-minute webinars were also offered to orient CHCs to the “bundle.”

This study utilizes three main strategies to further facilitate uptake of the ALL intervention, as embedded in the CVD Bundle. The strategies are described in detail in Table 1. In brief, they are the following:

**Table 1:** SPREAD-NET implementation components

Implementation support strategy	Description	Study arm 1	Study arm 2	Study arm 3
CVD risk management bundle implementation toolkit	• Overview of OCHIN’s DM/CVD QI bundle	X	X	X
	• Staff training/patient education materials			
	• Documents to support ALL implementation: underlying evidence			
	• How to use bundle tools in workflows			
	• Additional tools: posters, patient handouts, after-visit summary text			
	• Webinar on how to use the toolkit			
	• How to train your clinic staff to use the bundle			
	• How to implement practice changes, *e.g.*, Plan-Do-Study-Act (PDSA) cycles [110–118]			
	• Annual webinars with updates			
	• Relevant references and links			
In-person training, quarterly follow-up webinars	• 2-day training in Portland, Oregon: how to use the bundle, and how to train others to use it		X	X
	• Hands-on training in how to use bundle tools (e.g. panel management)			
	• Focus on building skills around change management techniques (*e.g.*, PDSA cycles)			
	• Quarterly webinars; topics chosen by clinics to enhance implementation skills			
On-site practice facilitation	• Up to 5 visits per clinic, including:			X
	­ Staff presentations			
	­ Coaching on presenting the tools to clinic staff, and using the tools in clinic workflows			
	­ Tailored problem-solving support to address barriers			
	­ Clinical questions fielded by practice facilitator			
	• Ongoing telephone/e-mail support as needed

1. CVD Bundle implementation toolkit (all arms). The toolkit [70] was designed to contain information that a clinic could use to implement the CVD bundle. It emphasizes aspects of the bundle related to cardioprotective prescribing (the content of the original ALL intervention), with instructions on generating feedback data and on how to understand and respond to the bundle’s automated alerts [34, 71–75]. It also includes suggestions for how clinics can encourage uptake of the CVD bundle. Key topics include orienting clinic staff to the CVD bundle’s medication guidelines, testimonials from other clinics that implemented similar guidelines, how to use the CVD bundle (alerts, roster tools, etc.) including pre-recorded webinars, tips on how to implement change, patient education materials (in English and Spanish), staff education materials, and slide decks to be used when training clinic staff.
2. In-person training, quarterly follow-up webinars (arms 2–3). The 2-day in-person training was held in Portland, Oregon; participants’ travel was paid for using study funds. In brief, trainees were taught how to use each aspect of the CVD bundle and the toolkit and how to teach their clinic colleagues about both. The first training day covered why the CVD bundle components were built, the evidence for their content, and a hands-on demonstration of the EHR tools. The second day included a panel session with providers and staff from CHCs that implemented ALL in our prior trial and in-depth hands-on training on change management techniques (*e.g.*, using the Plan-Do-Study-Act cycle for testing and refining changes and developing sample clinic workflows).
3. On-site practice facilitation (arm 3). A trained practice facilitator will go to the study sites and assist as needed with addressing any identified barriers to implementing the CVD bundle.

To further facilitate uptake of the ALL intervention, participating clinics were asked to identify a study “point person” and a “clinician champion.” We recommended selecting staff members to be point people if they were interested in diabetes care/cardiovascular disease prevention, considered credible/influential by other staff, passionate about care quality, and involved with clinic QI and/or change management activities. As seen in Table 2, clinics selected staff with varied backgrounds (technical, administrative, and clinical) to fill the point person role. Clinic champions will support the point person by serving as a resource for other providers wishing to understand the intervention and as a role model for other providers considering using the tools.

**Table 2:** Point people and clinic champions

Healthcare organization	Study arm	# of clinics	# of adult patients w/DM	# of point persons/clinic champions; role(s)
1	1	1	224	1: nurse care manager
2	1	1	547	1: physician’s assistant
3	1	2	1520	2: physician/medical director (both)
4	1	5	1781	2: clinical site specialist; patient population specialist
Arm total		9	4072	6 total
5	2	2	565	1: nurse practitioner
6	2	2	937	1: physician/medical director
7	2	2	246	1: physician/medical director
8	2	5	3320	3: epic EHR site specialists
Arm total		11	5060	6 total
9	3	1	556	1: clinical data analyst
10	3	2	493	1: nurse
11	3	2	871	2: pharmacy director; pharmacist
12	3	4	1740	4: director of performance improvement/population health; mental health/substance abuse counselor/social worker; patient advocate; office manager
Arm total		9	3660	8 total

### Study arms

The 29 study CHCs were randomized to one of three arms, each arm receiving a different level of implementation support (Table 1). Randomization was by healthcare organization, to avoid cross-clinic contamination within organizations, and weighted on number of diabetic patients, number of clinics, and urban/rural status.

### Study data, variables, and measurement

#### Variable selection

Variable selection was guided by Reach, Effectiveness, Adoption, Implementation, Maintenance (RE-AIM), a widely accepted framework for evaluating implementation of interventions [1, 76–78]. The primary independent variable is study arm. Dependent variables will represent measures of how effectively each strategy supports sustainable implementation of ALL. Our primary dependent variables will be change in rates of guideline-concordant prescribing of cardioprotective medication (ACE/ARBs, statins) among patients with diabetes. We will also assess process outcomes. Table 3 lists details measures and data sources.

**Table 3:** Study variable measurement

RE-AIM components	Measure	Data source
Reach: rate of guideline-appropriate prescribing of ACE/ARBs, statins [primary dependent variables]	(a) % clinic’s “indicated” patients with an active prescription (Rx issued in last year) for each indicated medication group (b) % of patients indicated for statins, on the correct dosage	Monthly EHR data
Effectiveness: proportion of patient BP, LDL “under control” (targeted by statins, ACE/ARBs, respectively) [secondary outcome]	% clinic’s “indicated” patients with: (a) last systolic BP <135, last diastolic BP <80 (b) last LDL <100	
Adoption: use of toolkit elements - Site participation/uptake rates - Staff participation/uptake rates overall/by staff role [Process outcome]	(a) % indicated patient encounters where (i) appropriate prescription given (ii) statin dosage corrected (b) rates of use of the roster tools (c) rates of responses to “charting alerts:” how often the recommended change is made in the chart (d) rates of responses to health maintenance alerts: data entered/recommended care provided (e) % staff attending relevant trainings (f) perceived value of patient/staff education materials	Monthly EHR data; qualitative data
Implementation: Compliance with, fidelity / adaptation to toolkit elements across and within sites	How toolkit elements are used: e.g., use of staff training materials, patient education materials, patient panel management rosters, other reminder tools; any adaptations made to these materials	Qualitative data
Maintenance: uptake of toolkit elements; primary, secondary outcome effects, over time	All measures at 1, 2, 3 years post-implementation	EHR, qualitative data

#### Baseline survey

We hope to describe characteristics of the clinics that have more or less implementation success at each level of support, to explore how such characteristics impact the ability to implement the tools in the CVD risk management bundle and how these factors impact the effectiveness of each implementation strategy [78–80]. To that end, we created two surveys to gather baseline practice characteristics: (i) the all-staff survey, designed to assess clinics’ readiness to change, and (ii) the clinic information form survey (CIF), designed to collect factual information about each clinic. While we will not have adequate power to conduct statistical assessments of association between survey findings and study outcomes, we intend to use these data to inform subsequent qualitative data collection and analysis and to generate hypotheses for future intervention analyses.

Survey item selection was informed by the literature on practices’ “readiness to change” [81–93] and input from CHC staff from our prior trial; surveys were piloted prior to finalizing. The final all-staff survey incorporates the adaptive reserve scale [94, 95] and the organizational readiness to change assessment instrument’s [96] context assessment subscale, as well as questions about perceived capacity to implement change, perceived QI needs, and staff demographics. The CIF covers questions about ownership, staffing, revenue, billing, and insurance characteristics and prior experiences with change. In addition, data from OCHIN records and the EHR will provide baseline information on EHR go-live dates, meaningful use attestation, and characteristics of the patient population. Study point people distributed and collected the all-staff surveys to all clinic staff (to ensure confidentiality staff were asked to seal their completed survey in a study-provided manila envelope and place a “confidential” sticker across the seal before returning to the point person); respondents received a $5 gift card after returning the completed survey. Point people also facilitated completion of the CIF by a clinic manager or QI coordinator.

#### Quantitative data

Our primary outcome of interest is clinic-level rates of guideline-concordant ACE/ARB and statin prescribing. We note that although the clinical success of ALL is dependent on patients taking recommended and prescribed medications, the first step—and SPREAD-NET’s primary outcome measure—is prescription of these medications. Quantitative data will capture medication orders/prescribing rates, changes in clinical outcomes (blood pressure and lipids), and decision support tool usage/responses. It will be extracted monthly from OCHIN’s EHR database using structured queries. Additional patient, provider, and encounter-level data will be extracted as necessary to control for confounding in our models. OCHIN’s EHR data is regularly cleaned and validated, ensuring data quality.

#### Qualitative data

We will use observation, interviews, and document review to explore the implementation process (e.g., how the toolkit was used/adapted in CHC workflows), how each support strategy affected implementation and sustainability, and facilitators and barriers to intervention uptake. Site visits will be conducted at clinics in all three study arms, purposively selected to maximize variation and optimize learning. Details are in Table 4.

**Table 4:** Qualitative data collection methods

Method	Type	Who/what	When	How often / many	Why
Interview	Check-in phone call	Point people (all)	Begin soon after implementation support begins; continue for remainder of study	Biweekly initial 6 months; monthly thereafter	Logistics of implementation (trainings); unresolved issues; implementation surprises, challenges, solutions
	Opportunistic interview	CHC providers and staff during in-person site visits	During site visits study years 2.5–4	As possible during 2-day site visits (12)	Personal experience with “bundle” tools; barriers and facilitators to use
	Semi-structured interview	CHC providers and staff during in-person site visits	During site visits study years 2.5–4	Minimum 4 interviews per clinic during 2-day site visits (12)	Perception of implementation/change process; barriers and facilitators to change; effectiveness of support strategy
	Phone interview	CHC providers and staff at clinics not visited in person	Study years 3–4	Minimum 2 interviews per clinic	Perception of implementation/change process; barriers and facilitators to change; effectiveness of support strategy.
	Debriefings	Study practice facilitator.	Study years 2.5–4	After each practice facilitation visit (45+)	Clinic-specific strengths and challenges surrounding implementation; effectiveness of support strategy
Observation	In-person observation	Clinic workflows, team interactions, patient encounters	During site visits years 2.5–4	Over course of 2-day site visits (12)	How intervention tools used in clinical care; insight into practice characteristics that influence uptake
Document review	Relevant archival data (e-mail conversations with CHC staff; clinic policies; etc.)	n/a	Throughout study	As available	Contextual information; insight into questions and negotiations surrounding implementation

### Analyses

#### Quantitative

For aim 1, we will use an interrupted time-series design and segmented regression to assess differences in the implemented intervention’s impact on patient health (effectiveness) and guideline-based prescribing (reach) [97–99]. Time in monthly intervals will be the unit of observation. The segmented regression model will estimate prescribing rates in the 18 months pre-implementation and how these change in the first year post-implementation. We will measure both the immediate effect of the intervention and estimate the effect across time. We will test whether the pre-post intervention change in trends in the rates over time differs across arms, as well as the immediate effect of the intervention. Aim 2 analyses will use the same approach, adding time points through 36 months post-intervention (maintenance). The time period variable will have three levels (pre-intervention, 12 months post-intervention, and 13–36 months post-intervention).

We will also conduct secondary analyses in which we will define thresholds of intervention impact. We will then assess the minimal amount of implementation support needed to achieve threshold results, using logistic regression models, with study arm predicting a binary outcome for threshold achieved by 12 months. These analyses will include an additional control group, using data from > 300 OCHIN CHCs not formally participating in the study, so that study arm has four levels of implementation support: none, low, medium, high. The “none” group will be the reference category, to assess if each of three implementation support approaches achieves the threshold target relative to no intervention.

#### Qualitative

Qualitative data collection and analysis will be concurrent and iterative, permitting us to identify salient constructs and knowledge gaps while implementation is ongoing, incorporate this knowledge into subsequent data collection, and guide adaptation of the support strategies [100–109]. The aim will be to understand implementation from the participants’ perspective, including barriers and facilitators, the extent to which toolkit elements are used or adapted (adoption), and the impact of each support strategy on implementation success (implementation). Attention will be paid to factors leading to a given practice excelling or struggling in response to the offered implementation support.

#### Costs

One key aspect of this study is the cost comparison for each strategy—an important consideration when replicating the intervention at future CHCs. We will identify the costs associated with each support strategy (such as programming, training clinic staff, other staff time needs, etc.). We will also estimate the incremental cost per additional unit of effect (e.g., cost per person gaining appropriate cardioprotective medication, cost per 100 additional patients receiving guideline-appropriate medication).

### Trial status

At the time of manuscript submission, the SPREAD-NET study is beginning year 2 of 5.

## Discussion

While substantial research has identified interventions that improve care quality, little is known about how to implement such interventions most effectively and efficiently in diverse care settings. Very little research has directly compared the effectiveness of different implementation strategies, especially in “real-world” clinics serving vulnerable populations; in fact, little prior research differentiated between the intervention being tested, and the methods used to implement it. The SPREAD-NET trial is designed to assess the level and type of support needed to successfully implement an EHR-based QI intervention in diverse CHCs and thus to fill these knowledge gaps about effective implementation in general and in the safety net setting in particular.

SPREAD-NET is poised to contribute important evidence about effective strategies for improving health care delivery by comparing approaches to helping CHCs implement an evidence-based “bundle” of decision support tools. As shown in Fig. 1, it fits within a larger line of research. First, the effectiveness of statins and ACE/ARBs at preventing CVD events in people with diabetes was demonstrated. Then, KP showed ALL’s effectiveness at implementing practice changes related to identifying patients indicated for these medications. Our first trial showed that ALL could be adapted for and implemented in CHCs, using concurrent, diverse implementation strategies. SPREAD-NET is designed to evaluate how much and what kind of implementation strategies are needed in CHCs; thus, it addresses the “final mile” involved in turning new scientific knowledge into practice. While SPREAD-NET uses a diabetes-focused intervention as our test case, findings are expected to apply to diverse interventions involving practice change implementation and implementation of evidence-based care guidelines.

Given that the field of D&I science is nascent, SPREAD-NET may serve as a model for future trials interested in identifying methods for encouraging uptake of EHR-based decision support, by clearly differentiating between the intervention and the methods used to implement it. This trial’s mixed methods design is important, as it will allow us to understand not just how often clinics acted on the targeted practice change but also why certain approaches did or did not work well in clinics with different characteristics. This protocol paper thus demonstrates methods for using mixed methods in implementation research.

In conclusion, the SPREAD-NET trial described here is an empirical example of the research that is needed to allow us to accelerate implementation timelines and reduce disparities in health care and health outcomes, especially in CHCs. It has the potential to contribute much-needed knowledge to the field of implementation science and to serve as a model for future research on how to implement effective interventions in diverse care settings.

## Abbreviations

ACE inhibitor: angiotensin-converting-enzyme inhibitor
ALL: aspirin, lovastatin, lisinopril
ARB: angiotensin receptor blocker
CHC: community health center
CVD: cardiovascular disease
EHR: electronic health record
KP: Kaiser Permanente
MI: myocardial infarction
QI: quality improvement
RE-AIM: Reach, Effectiveness, Adoption, Implementation, Maintenance
SPREAD-NET: Study of Practices Enabling Implementation and Adaptation in the Safety Net
